# The incidence, etiologies, outcomes, and predictors of mortality of acute liver failure in Thailand: a population-base study

**DOI:** 10.1186/s12876-019-0935-y

**Published:** 2019-01-28

**Authors:** Kessarin Thanapirom, Sombat Treeprasertsuk, Ngamphol Soonthornworasiri, Kittiyod Poovorawan, Roongruedee Chaiteerakij, Piyawat Komolmit, Kamthorn Phaosawasdi, Massimo Pinzani

**Affiliations:** 1 Division of Gastroenterology, Department of Medicine, Faculty of Medicine, Chulalongkorn University and King Chulalongkorn Memorial Hospital, Thai red cross society, Bangkok, 10700 Thailand; 2 Institute for Liver and Digestive Health, Royal Free Hospital, University College London, London, UK; 3 Department of Tropical Hygiene, Faculty of Tropical Medicine, Mahidol University, Bangkok, Thailand; 4 Department of Clinical Tropical Medicine, Faculty of Tropical Medicine, Mahidol University, Bangkok, Thailand; 5 Vichaiyut hospital and Medical Center, Bangkok, Thailand

**Keywords:** Acute liver failure, Epidemiology, Outcomes, Predictor, Population-based study

## Abstract

**Background:**

Acute liver failure (ALF) is uncommon but progresses rapidly with high mortality. We investigated the incidence, etiologies, outcomes, and predictive factors for 30-day mortality in patients with ALF.

**Methods:**

We conducted a population-based study of ALF patients hospitalized between 2009 and 2013 from the Thai Nationwide Hospital Admission database, which comprises 76% of all admissions from 858 hospitals across 77 provinces in Thailand. ALF was diagnosed using ICD-10 codes K72.0 and K71.11. Patients with liver cirrhosis were excluded.

**Results:**

There were 20,589 patients diagnosed with ALF during the study period with 12,277 (59.6%) males and mean age of 46.6 ± 20.7 years. The incidence of ALF was 62.9 per million population per year. The most frequent causes of ALF were indeterminate (69.4%), non-acetaminophen drug-induced (26.1%), and viral hepatitis (2.5%). Acetaminophen was the presumptive cause in 1.7% of patients. There were 5502 patients (26.7%) who died within 30 days after admission. One patient (0.005%) underwent liver transplantation. The average hospital stay was 8.7 ± 13.9 days, and the total cost of management was 1075.2 ± 2718.9 USD per admission. The most prevalent complications were acute renal failure (ARF)(24.2%), septicemia (18.2%), and pneumonia (12.3%). The most influential predictive factors for 30-day mortality were ARF (HR = 3.64, 95% CI: 3.43–3.87, *p* < 0.001), malignant infiltration of the liver (HR = 3.37, 95% CI: 2.94–3.85, *p* < 0.001), and septicemia (HR = 1.96, 95%CI: 1.84–2.08, *p* < 0.001).

**Conclusions:**

ALF patients have poor outcomes with 30-day mortality of 26.7% and high economic burden. Indeterminate etiology is the most frequent cause. ARF, malignant infiltration of the liver, and septicemia are main predictors of 30-day mortality.

## Background

Acute liver failure (ALF) is a clinical condition characterized by an acute deterioration of liver function resulting in encephalopathy and coagulopathy (International Normalized Ratio: INR ≥ 1.5), which occurs within 8–26 weeks of the onset of symptoms in patients without preexisting liver disease [1–3]. However, the definitions have variations in time course and etiology [1–4]. Acetaminophen and drug toxicity, viral hepatitis, autoimmune hepatitis, and indeterminate ALF are the most frequent causes of ALF. There are different predominant causes of ALF for each geographical area and ethnicity [5–7]. In the United States and European countries, acetaminophen toxicity is the predominant cause, while acute viral hepatitis infection is common in South and East Asia [5–10].

ALF is an uncommon condition, and the average incidence is approximately 5.5–6.2 people per milllion population per year [11, 12]. However, patients with ALF often rapidly progress to multiorgan failure and have a high mortality rate. Providing good supportive care in the intensive care unit, including cardiovascular monitoring, intracranial pressure (ICP) monitoring, and ventilator support, are important in the management of patients with ALF. Emergency liver transplantation remains the only definitive treatment for patients who do not achieve spontaneous recovery. However, a minority of ALF patients (7–18%) could receive emergency liver transplantation [13–15]. The in-hospital survival rates of patients with ALF treated with and without liver transplantation are 80–86% and 35–48%, respectively [9, 15].

Identifying the predictive factors of mortality is important and may lead to early intensive care treatment and consideration for emergency liver transplantation. Thus, this study aimed to determine the predominant causes, clinical outcomes, and prognostic factors of ALF. We conducted a nationwide population-based cohort study of patients with ALF during a 5-year period to evaluate the incidence, etiologies, clinical outcomes, economic burden, and predictive factors of 30-day mortality.

## Methods

### Study population and data acquisition

Data were obtained from the discharge records of hospitalized patients whose medical treatment expenses were covered by the Universal Coverage Scheme (UCS). The data contained 76% of all admissions from primary, secondary, and tertiary hospitals across 77 provinces in Thailand. The National Health Security Office (NHSO) reviewed and improved the quality of the 28,294,685 admission summary records in the database starting from 2009 until 2013, which aimed to examine the epidemiology and outcomes of patients hospitalized with gastrointestinal or liver diseases in Thailand. In the current study, we investigated the demographic data, co-morbidities, causes of ALF, therapeutic procedures, clinical outcomes, and medical expense of ALF patients enrolled in the NHSO database during these 5-year period.

The study enrolled patients who were hospitalized for ALF and diagnosed for the first time using codes K72.0 (Acute and subacute hepatic failure) and K 71.11 (Toxic liver disease with hepatic necrosis, with coma) of the International Classification of Diseases, 10th revision (ICD-10) from January 1, 2009 to December 31, 2013 according to the NHSO database. Patients who had preexisting liver cirrhosis (K74) were excluded. The diagnosis of ALF, co-morbidities, and treatment considerations were performed by attending physicians based on clinical data and laboratory investigations in each hospital. Patients with ALF were diagnosed based on the compatible criteria as the onset of hepatic encephalopathy occurring within 26 weeks of the onset of jaundice in patients with no preexisting cirrhosis [3].

Co-morbidities, etiologies, and complications were identified using following codes: cerebrovascular disease (I60–69), ischemic heart disease (I20–25), diabetes mellitus (E08–13), chronic obstructive pulmonary disease (J44), chronic kidney disease (N18–19), hepatitis A virus infection (B15), hepatitis B virus infection (B16, B18.0, and B18.1), hepatitis C virus infection (B17.1 and B18.2), hepatitis E virus infection (B17.2), autoimmune hepatitis (K75.4), vascular liver diseases (I82.0, K76.3 and K76.5), malignant neoplasms of liver and intrahepatic bile duct (C22), adverse effect of 4-aminophenol derivatives (T39.1), acute renal failure (ARF)(N17), septicemia (A41), pneumonia (J12–18), and urinary tract infection (N39). Patients were classified as indeterminate ALF when no etiological factor was identified. Therapeutic procedures were identified using ICD-9 procedure codes: liver transplantation (50.5), hemodialysis (39.95), antibiotic usage (99.21), blood transfusion (99.00–99.09), plasma exchange (99.71), ICP monitoring (01.10) and ventilator support (96.7). The additional ICD-10 external cause index was used to identify the etiologies of drug-induced ALF (T36-T65).

The study protocol was approved by the Institutional Review Board (IRB 113/58) of the Faculty of Medicine, Chulalongkorn University, Bangkok, Thailand. The study was conducted in accordance with the ethical principles of the Declaration of Helsinki under good clinical practice. The data were used by the Gastroenterological Association of Thailand (GAT) with an agreement from the NHSO.

### Definitions

Most government hospitals in Thailand are operated by the Ministry of Public Health and are classified into three levels. Primary hospitals are community hospitals at the district level and have capacities of 10–200 beds. Secondary hospitals are general hospitals in major districts and have capacities of 200–500 beds. Tertiary hospitals are regional hospitals in provincial centers and have capacities of at least 500 beds and specialist physicians.

### Statistical analysis

Categorical data are presented as numbers (percentages), and continuous data are shown as the mean ± standard deviation (SD). Comparison the difference between baseline characteristics, clinical outcomes and therapeutic procedures in patients who died and survived within 30 days is carried out using the chi-square test or Fisher’s exact test for categorical variables and the unpaired t-test or Mann-Whitney U test for continuous variables. Two-sided *P*-values < 0.05 were considered statistically significant. Cox proportional hazards regression analysis was used to identify independent predictive factors for 30-day mortality, and the results are shown as the hazard ratio (HR) and 95% confidence interval. All analyses were performed using SPSS software version 22.0 (IBM, New York city, NY, USA).

## Results

### Patient characteristics

Between January 2009 and December 2013, the incidence due to all-cause admissions was 5.6 million cases per year. Of these, a total of 159,061 admissions (or 21,165 admissions per year) were due to liver diseases, and 20,589 admissions (or 4117 admissions per year) were diagnosed with ALF and enrolled in this study. The annual admission rate for ALF was 0.07% of the total admissions per year and 19.4% of all admissions related to liver disease. According to the data from the National Statistical Office of Thailand (NSO), the estimated rate for the Thai population was 65.4 million people per year. The incidence of ALF in Thailand was 62.9 cases per million people per year in the present study. The mean age of hospitalized patients with ALF was 46.6 ± 20.7 years, and 12,277 patients were male (59.6%). Most of the patients were hospitalized in primary hospitals (*n* = 13,297, 64.6%) and in the Northeast part of Thailand (*n* = 8001, 38.9%). The patient’s baseline characteristics and co-morbidities were shown in Table 1.

**Table 1:** Baseline characteristics and demographic of patients hospitalized with acute liver failure during 2009 to 2013

Variables	Survived ≥ 30 days (*n* = 15,087)	Died within 30 days (*n* = 5502)	*p*-value
Age, years	44.8 ± 20.4	51.7 ± 20.6	< 0.001
Male, n (%)	8810 (58.4%)	3467 (63.0%)	< 0.001
Hospital level			
Primary, n (%)	9813(65.0%)	3484 (63.3%)	0.02
Secondary, n (%)	2789 (18.5%)	975 (17.7%)	0.21
Tertiary, n (%)	1106 (7.3%)	374 (6.8%)	0.19
Others, n (%)	1379 (9.1%)	669 (12.2%)	< 0.001
Residence by region			< 0.001
Central, n (%)	3187 (21.1%)	1254 (22.8%)	0.01
Northeast, n (%)	6075 (40.3%)	1926 (35.0%)	< 0.001
North, n (%)	1676 (11.1%)	775 (14.1%)	< 0.001
South, n (%)	1281 (8.5%)	523 (9.5%)	0.02
Others, n (%)	2865 (19.0%)	1024 (18.6%)	0.54
Co-morbidity			
Cerebrovascular disease, n (%)	111 (0.7%)	99 (1.8%)	< 0.001
Ischemic heart disease, n (%)	435 (2.9%)	376 (6.8%)	< 0.001
Diabetes mellitus, n (%)	1302 (8.6%)	584 (10.6%)	< 0.001
Chronic obstructive pulmonary disease, n (%)	304 (2.0%)	242 (4.4%)	< 0.001
Chronic kidney disease, n (%)	392 (2.6%)	291 (5.3%)	< 0.001
Etiology			
Hepatitis A virus, n (%)	16 (0.1%)	4 (0.1%)	0.50
Hepatitis B virus, n (%)	228 (1.5%)	139 (2.5%)	< 0.001
Hepatitis C virus, n (%)	84 (0.6%)	53 (1.0%)	0.001
Hepatitis E virus, n (%)	1 (0.00007%)	0	0.55
Acetaminophen, n (%)	323 (2.1%)	26 (0.5%)	< 0.001
Non-acetaminophen drugs, n (%)	4363 (28.9%)	1011 (18.4%)	< 0.001
Autoimmune, n (%)	7 (0.0005%)	8 (0.1%)	0.02
Vascular cause, n (%)	17 (0.1%)	21 (0.4%)	< 0.001
Malignant infiltration, n (%)	88 (0.6%)	272 (4.9%)	< 0.001
Other, n (%)	7 (0.0005%)	3 (0.1%)	0.82
Indeterminate, n (%)	10,250 (67.9%)	4049 (73.6%)	< 0.001

The etiologies of ALF in Thailand were indeterminate cause (69.4%), non-acetaminophen drug-induced (26.1%), viral hepatitis (2.5%), acetaminophen toxicity (1.7%), and other causes (0.3%). From the available recorded information (298 admissions), the main causes of non-acetaminophen drug-induced ALF were systemic antimycobacterial drugs (T37.1 and T36.6) (39.3%), systemic antibacterial antibiotics (T36.0–36.5, T36.8–36.9) (9.1%), and antiviral drugs (T37.5) (6.0%).

### Clinical outcomes and complications

The overall in-hospital and 30-day mortality rates of patients with ALF were 18.4% (*n* = 3792) and 26.7% (*n* = 5502), respectively. The mean length of hospital stay was 8.7 ± 13.9 days. The complications among patients with ALF were ARF (24.2%), septicemia (18.2%), pneumonia (12.3%), and urinary tract infection (4.2%). At the time of study enrollment, 19.5% received mechanical ventilation, and 2.4% of patients required renal replacement therapy. One patient (0.005%) underwent liver transplantation.

The mean cost per hospital admission for ALF was 1075.2 ± 2718.9 United States dollar (USD). The total medical expense of admission was 23 million USD per year. Of this amount, 4.2 million USD per year was spent but could not sustain the lives of patients. ALF patients who died within 30 days after admission had greater age (51.7 ± 20.6 vs. 44.7 ± 20.4 years, *p* < 0.001), a greater proportion of males (63.0% vs. 58.4%, *p* < 0.001), more co-morbidities (28.9% vs. 16.8%, *p* < 0.001), and more cases of indeterminate cause (73.6% vs. 67.9%) compared to patients who survived within 30 days (Table 1). In terms of clinical outcomes and interventions, the patients who died within 30 days after admission had shorter lengths of hospital stays, had significantly more complications (including ARF, septicemia, pneumonia, and urinary tract infections), and needed more blood transfusion, renal replacement therapy, plasma exchange and mechanical ventilator support (Table 2). No patient was treated with liver dialysis or monitored ICP in this database.

**Table 2:** Clinical outcomes, requirement of therapeutic procedures and medical expense in hospitalized patients with ALF during 2009–2013

	Survived ≥ 30 days (*n* = 15,087)	Died within 30 days (*n* = 5502**)**	*p*-value
Length of stay (days)	9.7 ± 15.6	6.1 ± 6.5	< 0.001
Complication			
Acute renal failure, n (%)	2062 (13.7%)	2923 (53.1%)	< 0.001
Septicemia, n (%)	1703 (11.3%)	2041 (37.1%)	< 0.001
Pneumonia, n (%)	1370 (9.1%)	1156 (21.0%)	< 0.001
Urinary tract infection, n (%)	585 (3.9%)	290 (5.3%)	< 0.001
Procedure during admission			
Liver transplantation, n (%)	1 (0.00007%)	0	0.55
Renal dialysis, n (%)	188 (1.2%)	309 (5.6%)	< 0.001
Ventilator, n (%)	1125 (7.5%)	2895 (52.6%)	< 0.001
Antibiotic usage, n (%)	381(2.53%)	156(2.84%)	0.22
Blood transfusion, n (%)	2470(16.4%)	2068(37.6%)	< 0.001
Plasma exchange, n (%)	3(0.02%)	8 (0.14%)	0.001
Costs per admission (USD)	959.4 ± 2884.1	1392.8 ± 2172.3	< 0.001

*USD* United States dollars

### Predictors for 30-day mortality

Multivariate Cox regression analysis was performed to identify the predictor for 30-day mortality by including potential factors from univariate Cox regression analysis. Table 3 presents the independent predictive factors for 30-day mortality among hospitalized patients with ALF. The three most commons factors associated with 30-day mortality were ARF (HR = 3.64, 95% CI: 3.43–3.87, *p* < 0.001), malignant infiltration of the liver (HR = 3.37, 95% CI: 2.94–3.85, *p* < 0.001), and septicemia (HR = 1.96, 95%CI: 1.84–2.08, *p* < 0.001). The ARF, malignant infiltration of the liver, and septicemia were observed in 53.1, 4.9 and 37.1% of patients who died in 30 days after admission compared to 13.7, 0.6 and 11.3% of those who survived.

**Table 3:** Predictive factor for 30-day mortality in patients with ALF

	Adjusted Hazard Ratio	95% CI	*p*-value
Primary hospital	1.08	1.02–1.14	0.01
Residence in central region	1.02	0.95–1.08	0.66
Age ≥ 60 years	1.29	1.22–1.37	< 0.001
Male	0.99	0.94–1.05	0.78
Co-morbidities			
Cerebrovascular disease	1.19	0.98–1.46	0.09
Ischemic heart disease	1.32	1.18–1.47	< 0.001
Diabetes mellitus	0.90	0.82–0.98	0.02
Chronic obstructive pulmonary disease	1.20	1.04–1.37	0.01
Chronic kidney disease	1.32	1.17–1.48	< 0.001
Etiologies			
Hepatitis A virus	0.85	0.32–2.27	0.75
Hepatitis B virus	1.44	1.21–1.72	< 0.001
Hepatitis C virus	1.37	1.04–1.81	0.03
Acetaminophen	0.47	0.32–0.69	< 0.001
Autoimmune hepatitis	1.35	0.67–2.70	0.40
Vascular cause	1.27	0.82–1.95	0.29
Malignant infiltration	3.37	2.94–3.85	< 0.001
Indeterminate	1.15	1.07–1.24	< 0.001
Complications			
Renal failure	3.64	3.43–3.87	< 0.001
Septicemia	1.96	1.84–2.08	< 0.001
Pneumonia	1.24	1.16–1.33	< 0.001
Urinary tract infection	0.75	0.66–0.84	< 0.001

## Discussion

The main finding of the current study was the incidence rate of ALF was 62.9 per million population per year in Thai population with a 30-day mortality rate after hospital admission of 26.7%. In addition, indeterminate etiology (69.4%) was the most common cause of ALF. Patients have higher risk of death within 30 days after admission if they have age ≥ 60 years, co-morbidities, malignant infiltration of the liver, Hepatitis B virus infection, Hepatitis C virus infection, indeterminate cause, and complications including ARF, septicemia and pneumonia.

The true incidence of ALF is unclear because there is scarce data from previous studies, which were mostly done in liver transplant units or tertiary referral centers. The estimated annual incidence of ALF is 5.5 cases per million population per year in the United States according to a population-based study and 6.2 cases per million population per year in Scotland in liver transplant centers [11, 12]. Our study included data from primary, secondary, and tertiary hospitals and showed a higher incidence rate of ALF in comparison to these studies. However, the incidence rate from our study is close to that reported by Ho et al., who found an annual incidence rate of 80.2 cases per million person-years in Taiwanese hospitalized patients with ALF [7]. These data might indicate that ALF is not a rare condition in some countries, including Thailand.

The etiology of ALF is an important factor for prognosis and management. The main causes of ALF are different in each area of the world. The predominant cause of ALF is acetaminophen in the United States, the United Kingdom, and Australia (36–43%) [16–18]. In Asian countries, the predominant causes are acute hepatitis virus infection, specifically hepatitis E virus in India (43.9%) [19] and hepatitis B virus in Japan (32.7%) and Taiwan (33%) [7, 20]. Indeterminate cause is the leading etiology of ALF in Scandinavian countries (43%), Chile (44%), and Sudan (38%) [21–23]. There were no previous data on etiology in adult patients with ALF in Thailand. However, data from a single tertiary care hospital reported that indeterminate cause (55%) was the main etiology among hospitalized Thai children with ALF [24]. Our study showed that indeterminate cause (69.4%) is the predominant etiology of ALF in Thailand. Of the established causes, non-acetaminophen drug toxicity (26.1%) was the most frequent. Thailand has a high burden of chronic hepatitis B infection with an estimated prevalence of 3.5% of the population [25]. However, our study showed that acute hepatitis B-induced ALF occurred in only 1.8% of patients, which might be underestimated because the diagnosis may be difficult by routine serology.

In terms of outcome after hospital admission, many ALF patients have complications such as infections and multiple organ failures, high mortality, and require high cost for management. Among the complications, ARF is the most frequent non-hepatic organ failure in patients with ALF (24.2%) in the current study. The overall in-hospital and 30-day mortality rates of hospitalized ALF patients were 18.4 and 26.7%, respectively. Previous single center study from the United Kingdom by R Marudanayagam et al. reported that the 30-day mortality was 26.7% without liver transplant and 13.7% with liver transplant in ALF patients who were admitted between January 1992 and May 2008. Additionally, a study by G Ostapowicz et al. enrolled ALF patients from 17 tertiary care liver centers around the United States between January 1998 and May 2001 and showed that the 30-day mortality rates of 57% without liver transplant and 16% with liver transplant [17, 26]. Acetaminophen overdose was the most common cause of ALF in both studies. Liver transplantation was done in 21.3 and 29% of ALF patients in the R Marudanayagam et al. study and the G Ostapowicz et al. study, respectively. Liver transplantation is considered a proven therapy for improving survival in patients with ALF. In our cohort, only one patient underwent liver transplantation due to the rapid progression of the disease and the limitations of the transplant system. From 2009 to 2013, Thailand had nine liver transplant centers, of which eight were located in the capital city and one was located in the northeastern region. According to the Thai Red Cross Organ Donation Center Database, there are approximately 87–158 donors and only 43–73 patients who received liver transplantation annually. Our information could be useful for improving health care policy for the management of ALF and promoting public awareness of organ donation.

Identifying patients who have poor survival outcomes is essential and will lead to prompt referral and consideration for emergency liver transplantation. Previous studies show that encephalopathy, extrahepatic organ failure (especially renal failure), and coagulopathy are poor prognostic factors for mortality [27]. In addition, short-term outcome varies according to etiology. The favorable prognosis was found with acetaminophen- and ischemic-induced ALF (60–80% short-term survival without transplantation) and poor prognosis with hepatitis B infection, indeterminate cause and drug other than acetaminophen-related ALF (17–25% short-term survival without transplantation) [17, 26]. Consistent with our study, acetaminophen is inversely associated with the 30-day mortality in patients with ALF. Patients with acetaminophen-induced ALF had favorable prognosis possibly due to the availability of anti-dote (N-acetylcysteine: NAC) and prompt therapy from history of drug usage. Previous study showed 66 % of patients had spontaneous recovery with NAC and supportive care therapy [17, 28]. Additionally, age ≥ 60 years, co-morbidities, malignant infiltration, Hepatitis B or C virus infection, indeterminate etiology, infectious complications including ARF, septicemia and pneumonia are independent predictors for 30-day mortality as well.

This study has some limitations. First, it was a retrospective study; there may have been some missing data and uncontrolled exposure. Furthermore, it relies on accurate data recordkeeping. Second, it might be interesting if we could analyze the difference of the model for end-stage liver disease (MELD) scores in patients with acute liver failure who died and survived within 30 days. However, some information about clinical findings and laboratory tests were lacking in the NHSO database, precluding us for estimating this score. Third, our study may not represent the true prevalence of indeterminate-ALF in Thailand; we hypothesized that several reasons led to a large number of indeterminate causes in our study, including (i) unavailability of further laboratory investigations in all hospitals especially in primary hospitals where the majority of ALF patients were hospitalized, (ii) co-existence of several potential etiologies possibly confounding the final diagnosis by attending physician, (iii) missing data and the inability to get a precise history due to encephalopathy. A recent publication shows that using diagnostic algorithms and expert review in combination with additional laboratory testing can verify etiologic diagnoses in 50% of patients initially considered as indeterminate-ALF [29].

## Conclusions

The incidence rate of ALF in Thailand is 62.9 cases per million population per year. Indeterminate causes are the major cause, and non-acetaminophen drug-induced is the most common identifiable cause of ALF cases. Patients with ALF have high 30-day mortality and high economic burden. Age, etiologies and complications of ALF are predictive factors of 30-day mortality after hospital admission.

## Abbreviations

95% CI: 95% confidence interval
ALF: Acute liver failure
ARF: Acute renal failure
CI: Confidence interval
GAT: Gastroenterological Association of Thailand
HR: Hazard ration
ICD: International Classification of Diseases
IRB: Institutional Review Board
NHSO: National Health Security Office
NSO: National Statistical Office of Thailand
SD: Standard deviation
UCS: Universal Coverage Scheme
USD: United States dollar
